# Addendum to “Remittance of primary cutaneous CD30+ lymphoproliferative disorder in a patient on adalimumab”

**DOI:** 10.1016/j.jdcr.2023.12.018

**Published:** 2024-01-17

**Authors:** Rosanne Ottevanger, Rutger C. Melchers, Koen D. Quint

**Affiliations:** Department of Dermatology, Leiden University Medical Center, Leiden, the Netherlands

**Keywords:** biologic agents, cutaneous T-cell lymphoma, inflammatory disease

*To the Editor:*

We would like to follow-up on our previously described case of a refractory cutaneous CD30-positive lymphoproliferative disorder (CD30+ LPD) that remitted after initiation of adalimumab for Crohn’s disease.^1^ After initial total remittance, the patient discontinued adalimumab therapy after 6 months because of the lack of improvement of his Crohn’s disease and switched to ustekinumab. A year later, patient developed neurologic symptoms with hypesthesia of the left side of his face, left foot and hand. A right-sided T2 hyperintense lesion of the basal ganglia was identified on magnetic resonance imaging of the brain. Two lumbar punctures were inconclusive and a positron emission tomography and computed tomography did not reveal any other abnormalities. Due to the neurologic symptoms the patient discontinued ustekinumab on his own initiative. When the patient was planned for a neuro-navigated biopsy, the preoperative stereotactic magnetic resonance imaging showed complete spontaneous resolution of the tumor, and neurologic symptoms had improved. However, the symptoms and the tumor recurred within a few months after cessation of ustekinumab, and a navigated biopsy revealed a cerebral localization of the known CD30+ T-cell lymphoma with an identical clone to the previous CD30+ LPD of the skin on the left leg that had remained in remission after the start of adalimumab. The biopsy of the tumor in the brain was complicated by 2 focal epileptic seizures and an aspiration pneumonia. The patient opted not to undergo any further diagnostics or therapies after shared decision making and palliative centered care was started. The patient died a few weeks later.

In short, we previously described a case of an initially refractory cutaneous CD30+ LPD that remitted with initiation of adalimumab for Crohn’s disease. After the adalimumab was discontinued and patient started ustekinumab, his primary cutaneous CD30+ LPD remained in remission. However, 1 year later, patient developed a systemic cerebral localization with an identical clone to the primary cutaneous lesion, without any signs of lymphadenopathy. Systemic lymphomas are described after biologic therapy, but systemic localizations of cutaneous CD30+ LPD in complete cutaneous remission have not been described before.^2^ Whether this clinical course is associated with biologic therapy cannot be determined. Consideration for biologic therapy in the setting of cutaneous lymphoma should be weighed carefully, despite our previous report of an initial favorable response of refractory cutaneous CD30+ LPD to adalimumab. Future studies are needed to focus on the role of biologics and primary cutaneous CD30+ LPDs.^3^

## Conflicts of interest

None disclosed.
